# Prediabetes and structural brain abnormalities: Evidence from observational studies

**DOI:** 10.1002/dmrr.3261

**Published:** 2019-12-19

**Authors:** Jian‐Bo Zhou, Xing‐Yao Tang, Yi‐Peng Han, Fu‐qiang Luo, Marly Augusto Cardoso, Lu Qi

**Affiliations:** ^1^ Department of Endocrinology, Beijing Tongren Hospital Capital Medical University Beijing China; ^2^ Beijing Tongren Hospital Capital Medical University Beijing China; ^3^ Department of Nutrition, School of Public Health University of São Paulo São Paulo Brazil; ^4^ Department of Epidemiology, School of Public Health and Tropical Medicine Tulane University New Orleans LA

**Keywords:** glycosylated haemoglobin, meta‐analysis, prediabetes, structural brain abnormalities

## Abstract

Type 2 diabetes mellitus has been linked to structural brain abnormalities, but evidence of the association among prediabetes and structural brain abnormalities has not been systematically evaluated. Comprehensive searching strategies and relevant studies were systematically retrieved from PubMed, Embase, Medline and web of science. Twelve articles were included overall. Stratified analyses and regression analyses were performed. A total of 104 468 individuals were included. The risk of infarct was associated with continuous glycosylated haemoglobin (HbA_1c_) [adjusted odds ratio (OR) 1.19 (95% confidence interval [CI]: 1.05‐1.34)], or prediabetes [adjusted OR 1.13 (95% CI: 1.00‐1.27)]. The corresponding ORs associated with white matter hyperintensities were 1.08 (95%CI: 1.04‐1.13) for prediabetes, and 1.10 (95%CI: 1.08‐1.12) for HbA_1c_. The association was significant between the decreased risk of brain volume with continuous HbA_1c_ (the combined OR 0.92, 95% CI: 0.87‐0.98). Grey matter volume and white matter volume were inversely associated with prediabetes [weighted mean deviation (WMD), −9.65 (95%CI: −15.25 to −4.04) vs WMD, −9.25 (95%CI: −15.03 to −3.47)]. There were no significant association among cerebral microbleeds, hippocampal volume, continuous total brain volume, and prediabetes. Our findings demonstrated that (a) both prediabetes and continuous HbA_1c_ were significantly associated with increasing risk of infarct or white matter hyperintensities; (b) continuous HbA_1c_ was associated with a decreased risk of brain volume; (c) prediabetes was inversely associated with grey matter volume and white matter volume. To confirm these findings, further studies on early diabetes onset and structural brain abnormalities are needed.

## 1 INTRODUCTION

Type 2 diabetes has been described as a risk factor for cerebrovascular disease, including infarcts, dementia,^1^ depression,^2^ cognitive impairment^3^ and stroke.^4^ It has also been thought to be associated with brain diseases, such as white matter hyperintensities (WMHs), hippocampus volume^5^ and brain atrophy,^6^ measured by magnetic resonance imaging (MRI).^7^ Structural brain abnormalities, such as lacunar infarction and intracerebral haemorrhage, easily progress to stroke.^8^, ^9^ Most researchers believe that they appear because of the microvascular complications.^10^ A dose‐response effect of chronic hyperglycemia on microvascular complications exists, which is termed the metabolic memorial effect.^11^

Prediabetes is the early stage of diabetes development that includes the impaired fasting glucose (IFG) and impaired glucose tolerance (IGT) conditions, and can be diagnosed using fasting glycaemia and 2 hour glucose load test according to World Health Organization (WHO) definitions.^12^ Evidence proves that glycosylated haemoglobin (HbA_1c_) can also be a measure of prediabetes.^13^ Indeed, there is strong evidence that suggests prediabetes is associated with an increasing risk of stroke,^14^ dementia^15^ and cognitive impairment.^16^ The relationship between prediabetes and different kinds of structural brain abnormalities is still controversial,^17^ although similar micro‐ and macrovascular dysfunction were shown to be present.^18^ In contrast, in an elderly study population, there was no relationship reported between prediabetes and lacunar infarcts (LIs), cerebral microbleeds (CMBs), WMHs, or smaller brain volumes.^19^ These inconsistent findings may arise from different definitions and thresholds for prediabetes as well as small sample sizes. Becoming aware of the relationship between prediabetes and structural brain abnormalities would be beneficial to the prevention and treatment of related brain diseases.^20^

To our knowledge, this is the first meta‐analysis to examine the available evidence of a relationship between prediabetes status and risk for structure brain abnormalities. Considering previous inconsistent results, a meta‐analysis to explore the association of prediabetes with the risk of structure brain abnormality may help to clarify this issue. Thus, we searched the related articles and performed a systematic review and comprehensive analysis.

## 2 MATERIALS AND METHODS

### 2.1 Study inclusion

The databases PubMed, Embase, Medline and Web of Science were searched for relevant studies published until May 2019. The included terms ‘glucose blood level’, ‘haemoglobin a1c’, ‘blood fluctuation’, ‘impaired glucose tolerance’, ‘blood glucose’, ‘prediabetes’, ‘white matter hyperintensities’, ‘brain atrophy’, ‘brain haemorrhage’, ‘lacunar stroke’, ‘lacunar infarct’, ‘total cerebral brain volume’, ‘white hyperintensity volume’, ‘brain infarction’, ‘brain infarct’, ‘cerebral microbleed’, ‘hippocampal hyperintensity volume’, ‘magnetic resonance imaging’ were used alone and in combination. The search strategy was supplemented by inspecting the references of the included articles. This report was conducted according to the Meta‐analysis Of Observational Studies in Epidemiology^21^ and the Preferred Reporting Items for Systematic Reviews and Meta‐Analysis^22^ guidelines.

### 2.2 Inclusion and exclusion criteria

Studies were considered for inclusion using the following criteria: (a) was an original article recently published in English, (b) defined prediabetes or impaired glucose tolerance (IGT) or impaired fasting glucose (IFG) and any imaging appearances of structural brain abnormalities clearly, (c) investigated either continuous or categorial structural brain abnormalities, (d) measured the structural brain abnormalities using Magnetic Resonance Imaging (MRI), (e) used physical diagnosis of prediabetes, IGT, IFG or continuous HbA_1c_, (f) provided quantitative measures of the association between prediabetes, IGT or IFG and any type of structural brain abnormalities, and their 95% confidence intervals (CIs), (g) used cross‐sectional, case‐control or cohort epidemiological study designs. Exclusion criteria were as follows: (a) the publication was a review, case report, animal study or letter to the editor, (b) the publication did not clearly define clinical outcomes, (c) the authors could not provide valid data after being contacted, (d) the publication provided duplicated data.

For this meta‐analysis, only cohort studies about the association between continuous HbA_1c_ with any type of structural brain abnormalities and cross‐sectional studies about the relationship between prediabetes and any type of structural brain abnormalities were included.

### 2.3 Data extraction and quality assessment

Searching and screening were completed independently by two reviewers (X.Y.T. and Y.P.H.) and any discrepancies were resolved by discussion. Two investigators (X.Y.T. and Y.P.H.) independently search the extracted the data from the enrolled studies. Two investigators (X.Y.T. and F.Q.L.) independently utilised the Newcastle‐Ottawa Quality Assessment Scale criteria (NOS)^23^ to assess the risk of bias. We rated the quality of the studies by awarding stars following the guidelines of the Newcastle‐Ottawa Scale. Three factors were considered for the included cohort studies: selection, comparability, and outcome; for cross‐sectional studies, selection, comparability and exposure were considered. If there was disagreement, the investigators discussed the study with the other authors to arrive at a consensus.

### 2.4 Statistical analysis

Heterogeneity between studies was evaluated by *I* ^2^ metric, and the variance between studies by Tau^2^. Random‐effects models were performed if *I* ^2^ > 50% and fixed‐effects models were performed if *I* ^2^ ≤ 50%. Odds ratio (OR) as measure of association across all studies were pooled in categorial data defined as meeting the diagnostic criteria or not. Mean difference was pooled by using the inverse variance weighting method and presented as weighted mean difference (WMD) with 95% confidence interval in continuous data. If studies had both unadjusted and covariate‐adjusted ORs, we chose the latter. We regarded IGT and IFG as prediabetes.

In categorial data, for studies providing β, we convert this to OR. Potential publication bias was evaluated by Egger's asymmetry test.^24^ *P*‐values were two‐tailed, and *P* < .05 was considered statistically significant. The statistical analyses were performed with STATA version 12.0 (Stata Corporation, College Station, TX).

## 3 RESULTS

### 3.1 Study selection and study characteristics

**Figure 1:**
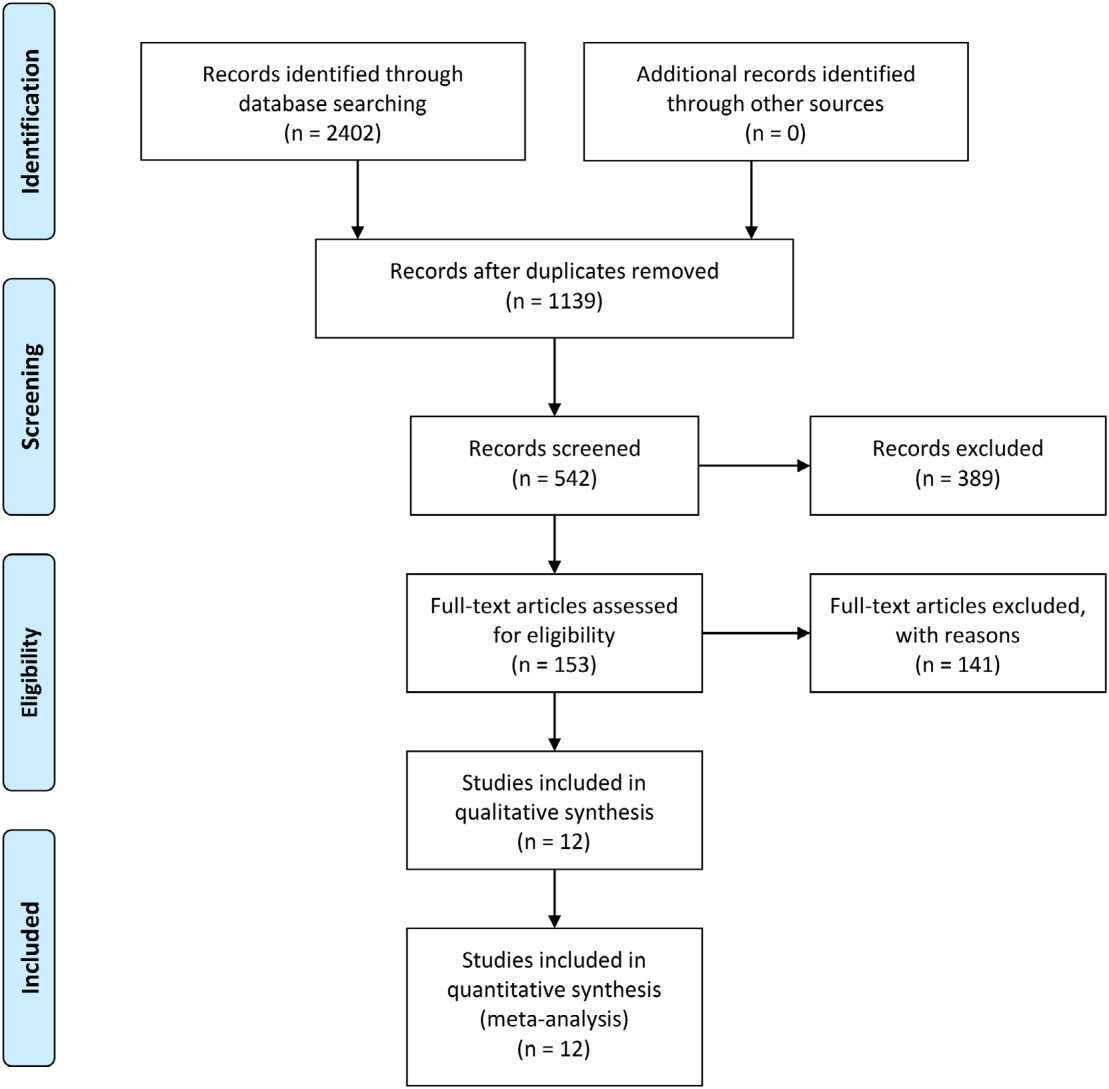
Results of systematic literature search

The search strategy identified 2402 potentially relevant records, of which 1263 were excluded as they were duplicates. The remaining 1139 manuscripts were subject to title and abstract screening. A further 986 publications were removed, as they were reviews, letters or conference abstracts and unrelated studies. Therefore, 153 articles were eligible for full‐text review and data assessment (Figure 1). Finally, 141 articles were excluded because they were animal studies (n = 9), unable to provide information (n = 120) or without a full publication (n = 12). The remaining 12 studies were enrolled in the meta‐analysis.^17^, ^19^, ^20^, ^25^, ^26^, ^27^, ^28^, ^29^, ^30^, ^31^, ^32^, ^33^ There were 4 prospective cohort studies,^26^, ^27^, ^29^, ^31^ 6 cross‐sectional studies^19^, ^25^, ^28^, ^30^, ^32^, ^33^ and 2 with both cohort and cross‐sectional data studies.^17^, ^20^ For continuous HbA_1c_, 4 cohort studies investigated different structural brain abnormalities^17^, ^29^, ^30^, ^31^: 2 involved white matter hyperintensities,^30^, ^31^ 3 involved infarct,^17^, ^30^, ^31^ and 2 involved brain volume^29^, ^31^). For prediabetes, 8 cross‐sectional studies were chosen:^17^, ^19^, ^20^, ^25^, ^28^, ^30^, ^32^, ^33^ 5 studies involved infarct,^17^, ^19^, ^30^, ^32^, ^33^ 3 studies involved white matter hyperintensities,^19^, ^20^, ^30^ 3 studies involved grey matter volume (including all kinds of grey matter),^19^, ^20^, ^30^ 2 studies involved hippocampal volume,^19^, ^20^ 2 studies involved white matter volume,^20^, ^30^ 2 studies involved total brain volume,^19^, ^20^ and 2 studies involved cerebral microbleeds.^19^, ^30^ Three cross‐sectional studies^17^, ^28^, ^30^ provided continuous data of the relationship between prediabetes and structural brain abnormalities (3 involved white volume and all kinds of grey matter volume,^17^, ^28^, ^30^ 2 involved total brain volume^28^, ^30^). Table 1 and Table S1 provide an overview of the 12 eligible studies. Tables S2~S3 summarise the data reported in the studies synthesised by the meta‐analyses.

**Table 1:** Characteristics of the studies on the relationship between prediabetes and structural brain abnormalities

Source	Year	Location	Study design	Population (n) and age (years) of participants
Hirabayashi et al	2016	The town of Hisayama in Japan	Cross‐sectional study	Normal: 367 participants aging ≥65 years old. IFG: 53 participants aging ≥65 years old. IGT: 280 participants aging ≥65 years old.
Imano et al	2018	Ikawa town in Japan, the Minami‐Takayasu district in Yao City, Noichi town in Japan, and Kyowa town in Japan.	Prospective cohort study	Normal: 2072 men and 4171 women aging 40‐74 years old. Prediabetic: 317 men and 279 women aging 40‐74 years old.
Jin et al	2019	Tangshan city, China	Prospective cohort study	Normal: 78721 participants aging18‐98 years old IFG: 6331 participants aging 18‐98 years old
Walsh et al	2018	Australia	Cross‐sectional study	NFG: 353 participants aging 53‐78 years old IFG: 95 participants aging 53‐78 years old
Marseglia et al	2019	Sweden	Prospective cohort study and cross‐sectional study	Diabetes free: 1557 participants aging ≥60 years old Prediabetes:947 participants aging ≥60 years old
Enzinger et al	2005	Australia	Prospective cohort study	201 participants aging 50‐75 years old
Agtmaal et al	2018	Southern part of the Netherlands	Cross‐sectional study	NGM: 1373 participants aging 40‐75 years oldPrediabetes: 347 participants aging 40‐75 years old
Exalto et al	2014	VU university Medical Centre in Netherland	Prospective cohort study	274 participants aging ≥45 years old, of which 158 were men.
Schneider et al	2017	Four U.S. communities: Washington County, Maryland; Forsyth County, North Carolina; the suburbs of Minneapolis, Minnesota; and Jackson, Mississippi	Cross‐sectional study	No diabetes: 597 participants aging 45‐64 years old Prediabetes: 514 participants aging 45‐64 years old
Reitz et al	2016	The institutional review boards Columbia University Medical Center and Columbia University Health Sciences and the New York State Psychiatric Institute	Cross‐sectional: longitudinal cohort study	NGT: 115 participants aging ≥65 years old Prediabetes: 224 participants aging ≥65 years old
Saczynski et al	2009	The Age Gene/Environment Susceptibility–Reykjavik Study	Cross‐sectional study	Normoglycemic: 2327 participants mean aging 76 years old IFG: 1599 participants mean aging 76 years old
Eastwood et al	2015	North‐west London	Cross‐sectional study	European ethnicity: 682 participants aging 58‐85 years old, of which 153 were men South Asian ethnicity: 520 participants aging 58‐85 years old, of which 78 were men

Abbreviations: IGT, impaired glucose tolerance; IFG, impaired fasting glucose; NFG, normal fasting glucose; NGT: normal glucose tolerance.

### 3.2 Quality assessment

Quality assessment results of the studies all received 6 to 8 stars using the Newcastle‐Ottawa scale (NOS) evaluation tool. The quality of the included studies was high. (Table S4).

## 4 META‐ANALYSIS RESULTS

### 4.1 Continuous HbA_1c_ vs categorial structural brain abnormalities

There were four sets of data on white matter hyperintensities (Q = 3.80, *I* ^2^ = 21.0%, *P* = .284), three sets on infarct (Q = 3.07, *I* ^2^ = 34.9%, *P* = .215) and two sets on brain volume (Q = 0.51, *I* ^2^ = 0.0%, *P* = .475). Thus, the fixed effect model was selected for combined analysis.

**Figure 2:**
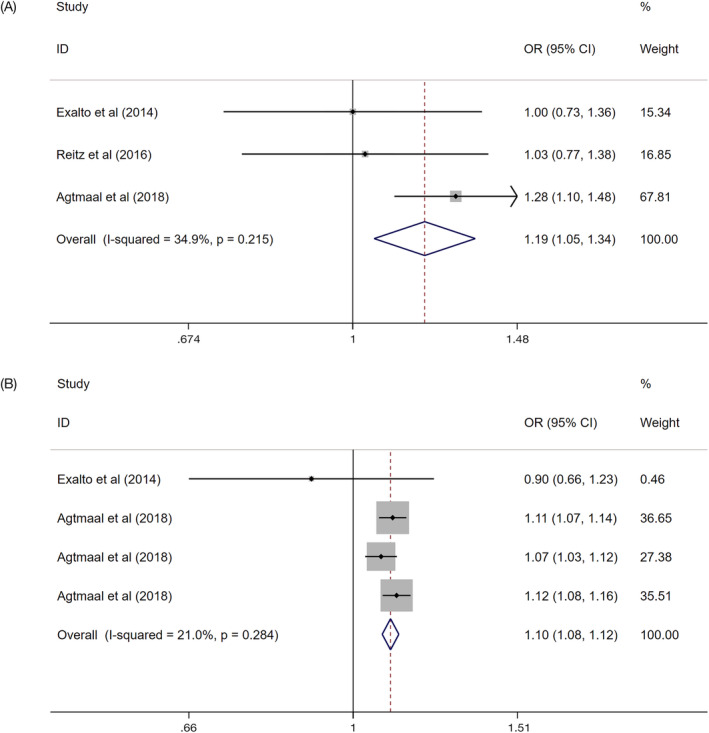
The association between continuous HbA_1c_ with infarct, A, and white matter hyperintensities, B. A, The association between continuous HbA_1c_ with infarct. B, The association between continuous HbA_1c_ with white matter hyperintensities. Where *I* ^2^ is the variation in effect estimates attributable to heterogeneity, overall is the pooled fixed effect estimate of all studies. Subtotal is the pooled fixed effects estimate of sub‐group analysis studies. Weights are from fixed‐effects analysis. Percentage of weight is the weight assigned to each study, based on the inverse of the within‐ and between‐study variance. The size of the grey boxes around the point estimates reflects the weight assigned to each study. The summarized studies were adjusted for age, sex and BMI. Abbreviation: OR, odds ratio

**Figure 3:**
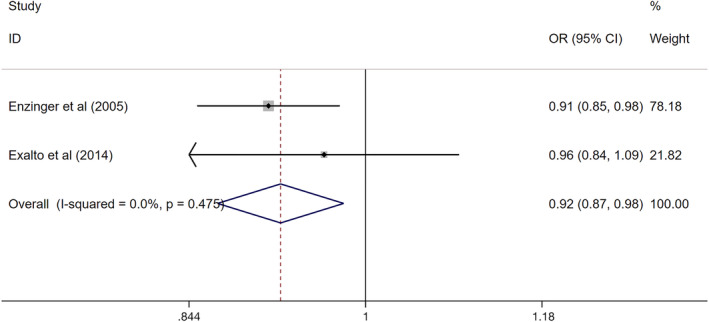
The association between continuous HbA_1c_ with brain volume. Where *I* ^2^ is the variation in effect estimates attributable to heterogeneity, overall is the pooled fixed effect estimate of all studies. Subtotal is the pooled fixed effects estimate of sub‐group analysis studies. Weights are from fixed‐effects analysis. Percentage of weight is the weight assigned to each study, based on the inverse of the within‐ and between‐study variance. The size of the grey boxes around the point estimates reflects the weight assigned to each study. The summarized studies were adjusted for age, sex and BMI. Abbreviation: OR, odds ratio

The pooled effect value of the relationship between infarct and continuous HbA_1c_ was statistically significant (Z test, Z = 2.79, *P* = .005, the combined OR 1.19, 95% CI: 1.05‐1.34). (Figure 2A) A similar positive association was obtained between white matter hyperintensities and continuous HbA_1c_ (Z test, Z = 8.82, *P* = .000, the combined OR 1.10, 95% CI: 1.08‐1.12). (Figure 2B) In contrast, brain volume was decreased with continuous HbA_1c_ (Z test, Z = 2.64, *P* = .008, the combined OR 0.92, 95% CI: 0.87‐0.98). (Figure 3).

### 4.2 Prediabetes vs categorical structural brain abnormalities

There were nine groups of data on infarct (Q = 11.03, *I* ^2^ = 27.5%, *P* = .200), five groups on white matter hyperintensities (Q = 3.13, *I* ^2^ = 0.0%, *P* = .537), three groups on cerebral microbleeds (Q = 3.99, *I* ^2^ = 49.9%, *P* = .136), three groups on grey matter volume (Q = 0.48, *I* ^2^ = 0.0%, *P* = 0.490) and two on hippocampal volume (Q = 0.98, *I* ^2^ = 0.0%, *P* = 0.323). Therefore, the fixed effect model was selected for further analysis.

**Figure 4:**
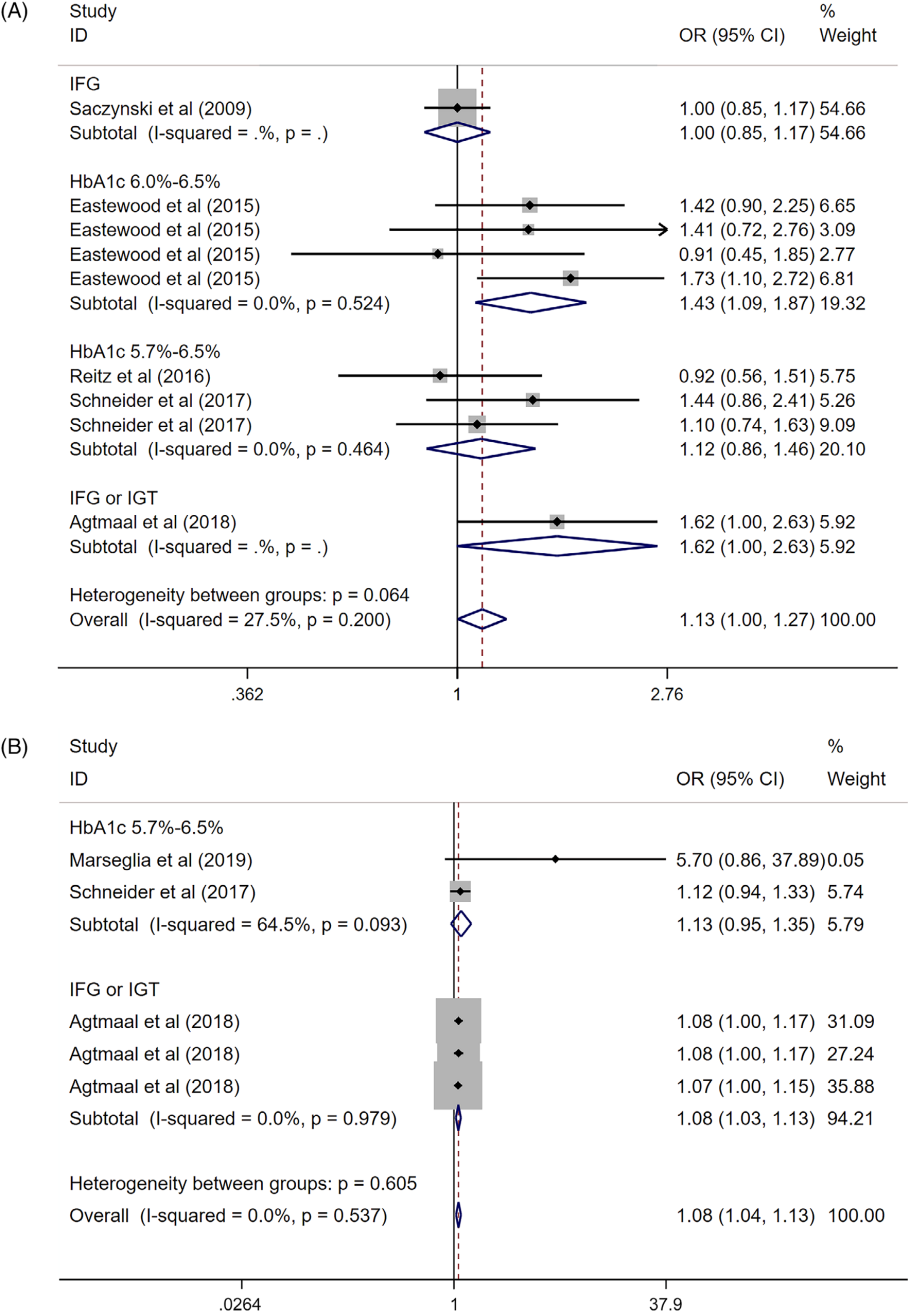
The association between prediabetes with infarct, A, and white matter hyperintensities, B. A, The association between prediabetes with infarct. B, The association between prediabetes with white matter hyperintensities. Where *I* ^2^ is the variation in effect estimates attributable to heterogeneity, overall is the pooled fixed effect estimate of all studies. Subtotal is the pooled fixed effects estimate of sub‐group analysis studies. Weights are from fixed‐effects analysis. Percentage of weight is the weight assigned to each study, based on the inverse of the within‐ and between‐study variance. The size of the grey boxes around the point estimates reflects the weight assigned to each study. The summarized studies were adjusted for age, sex and BMI. Abbreviations: OR, odds ratio; WMD, weighted mean deviation

The relationship between infarct and prediabetes remained constant (Z = 2.00，*P* = .046, the overall OR = 1.13, 95%CI: 1.00‐1.27). According to the different methods of prediabetes' diagnosis, we divided the data into four groups: HbA_1c_ 5.7%‐6.5%, HbA_1c_ 6.0%‐6.5%, IFG or IGT and IFG. Subgroup analysis revealed that non‐significant association was found in both HbA_1c_ 5.7%‐6.5% and IFG groups [the overall OR = 1.12 (95%CI: 0.86‐1.46) vs the overall OR = 1.00 (95%CI: 0.85‐1.17)]. However, prediabetes diagnosed by using HbA_1c_ 6.0%‐6.5% and IFG or IGT were also associated with infarct [(Z = 2.59, *P* = .010, the overall OR = 1.43, 95% CI: 1.10‐1.87), (Z = 1.95, *P* = .05, the overall OR = 1.62, 95% CI: 1.00‐2.63), respectively]. (Figure 4A).

White matter hyperintensities were related to prediabetes (Z = 3.70，*P* = .000, the overall OR = 1.08, 95%CI: 1.04‐1.13). In the subgroup analysis, diagnosis according to IFG or IGT revealed a positive result with overall OR = 1.08 (95%CI: 1.03‐1.13), while no statistical significance was found by using HbA_1c_ 5.7%‐6.5%, giving an overall OR = 1.13 (95%CI: 0.95‐1.35). (Figure 4B).

The relationship between cerebral microbleeds and prediabetes was not significant (Z = 0.15，*P* = .878, the overall OR = 0.98, 95%CI: 0.77‐1.26) (Figure 1A). Similar results were observed in both grey matter volume and hippocampal volume (Z = 0.86，*P* = .388, the overall OR = 1.05, 95%CI: 0.94‐1.17; Z = 0.20,*P* = .84, the overall OR = 1.01, 95%CI: 0.92‐1.11, respectively). (Figure 1B,C).

### 4.3 Prediabetes vs continuous structural brain abnormalities

There were three references for white matter volume (Q = 1.93, *I* ^2^ = 0.0%, *P* = .382), 3 references for grey matter volume (Q = 2.71, *I* ^2^ = 26.3%, *P* = .257) and 2 references for total brain volume (Q = 0.44, *I* ^2^ = 0.0%, *P* = .509) modelled as a continuous variable. The fixed effect model was used for combined analysis.

**Figure 5:**
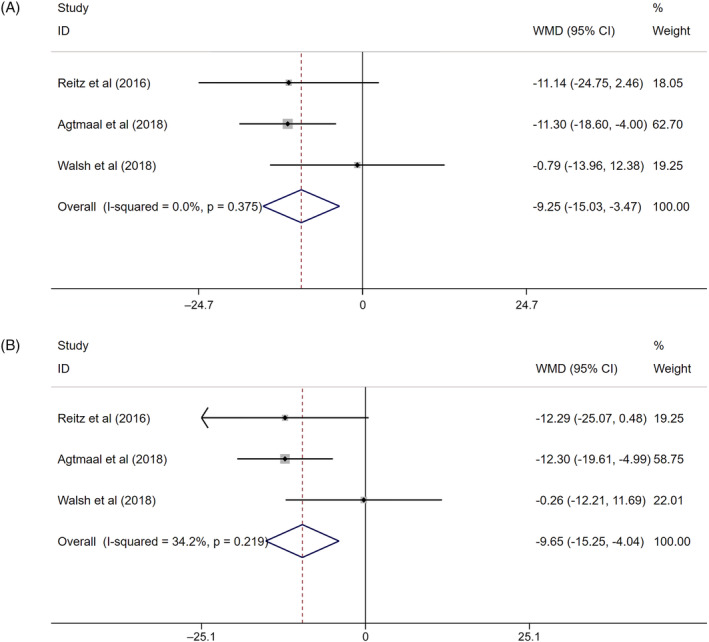
The association between prediabetes with continuous white matter volume, A, and continuous grey matter volume, B. A, The association between prediabetes with continuous white matter volume. B, The association between prediabetes with continuous grey matter volume. Where *I* ^2^ is the variation in effect estimates attributable to heterogeneity, overall is the pooled fixed effect estimate of all studies. Subtotal is the pooled fixed effects estimate of sub‐group analysis studies. Weights are from fixed‐effects analysis. Percentage of weight is the weight assigned to each study, based on the inverse of the within‐ and between‐study variance. The size of the grey boxes around the point estimates reflects the weight assigned to each study. The summarized studies were adjusted for age, sex and BMI. Abbreviation: WMD, weighted mean deviation

The pooled WMD for white matter volume in prediabetes was −9.65 (95%CI: −15.25 to −4.04) (Figure 5A), indicating an inverse correlation. Similarly, grey matter volume was inversely associated with prediabetes [overall WMD, −9.25 (95%CI: −15.03 to −3.47)] (Figure 5B). However, there was no relationship between total brain volume and prediabetes [overall WMD, −9.09, (95%CI: −22.99 to 4.81)] (Figure 1D).

### 4.4 Publication bias

According to the Cochrane Handbook version 5.1.0,^34^ as a rule of thumb, tests for funnel plot asymmetry should be used only when there are enough studies included in the meta‐analysis, because when there are fewer studies the power of the tests is too low to distinguish chance from real asymmetry. In this study, the *P* value of the Egger test was >.05 (*P* = .095) for the relationship between prediabetes and infarct, indicating no significant bias among them. The funnel figure of these studies showed a symmetrical inverted distribution, which is consistent with the results of Egger test (Figure 2).

## 5 DISCUSSION

In this study, we analysed the accumulated evidence to explore the relationship between prediabetes or continuous HbA_1c_ and structural brain abnormalities from observational studies. Our results indicate that (a) both prediabetes and continuous HbA_1c_ were significantly associated with an increased risk for infarct or white matter hyperintensities; (b) continuous HbA_1c_ was negatively correlated with brain volume; and (c) prediabetes was inversely associated with grey matter volume and white matter volume.

Although valuable information regarding relationships between prediabetes or continuous HbA_1c_ and different kinds of structural brain abnormalities was presented by our accumulated evidence analyses, important study strengths and limitations merit mention. The strengths of our study included choosing a consistent adjustment for multiple confounders including sex, age and other risk factors of cardiovascular diseases, such as smoking, education and economy. Furthermore, a very thorough search strategy was developed and a very broad search including all types of studies was conducted.

There were still some limitations to this research. (a) The development of structural brain abnormalities may start in middle age,^35^ and we failed to pursue further analysis on this point as most articles focus on the elderly population. (b) The heterogeneity of results may be due to differences in race, blood glucose measurement and the method of using and explaining the MRI. Most of the selected studies had the small sample size and some articles did not provide necessary information, so it was difficult for us to further analyse the heterogeneity. (c) This article analysed the relationship between some kinds of structural brain abnormalities and continuous HbA_1c_ or prediabetes. Apart from these, there were also some other type of structural brain abnormalities which could not be analysed due to limited dataset, such as cerebrospinal fluid (CSF),^30^ thalamus volume and corpus callosum volume.^28^ (d) In clinical practice, different kinds of structural brain abnormalities may appear together and their interactions with each other is unclear. A more comprehensive analysis was not possible because of different combinations considered in the studies. (e) The diagnostic criteria of prediabetes were inconsistent among the enrolled studies. Unfortunately, subgroup analysis was not possible due to small number of studies.

## 6 THE POSSIBLE MECHANISMS

Prediabetes stages can influence changes in cerebral energy homeostasis, may cause inflammation, and impact the vasculature at the arterial and the capillary level.^36^ Elevated blood sugar leads to the endothelial dysfunction of the cerebral or microcirculatory system,^18^ thus contributing to cerebral perfusion deficits and developing into chronic ischemia of the brain tissue.^37^ Disruption of the blood‐brain barrier can occur because of the production of reactive oxygen species and limited antioxidant defences due to hyperglycemia.^38^ In earlier stages of prediabetes, perivascular edema can cause cumulative insidious perivascular tissue damage, eventually resulting in the rarefaction and demyelination of white matter in the pathology, which can induce structural abnormalities and be shown as WMH on MRI.^39^ Neurodegeneration and brain atrophy can be caused by cerebral insulin resistance, which may impair regional glucose metabolism and disrupt the intracellular release, and extracellular clearance, of b‐amyloid.^30^

There is also evidence that other structural brain abnormalities associated with prediabetes exist. In a population‐based cohort study by Agtmaal et al., a significant association was found between prediabetes and cerebrospinal fluid (CSF) (β = 3.9, 95% CI = 0.3‐7.6), and a similar association was observed with continuous HbA_1c_ (β = 0.09, 95% CI = 0.06‐0.11).^30^

### 6.1 Prospects

Future studies should pay more attention to the relationship between prediabetes or continuous HbA_1c_ and structural brain abnormalities according to different age groups. More systematic literature reviews on the association between prediabetes and structural brain abnormalities should be down to enrich the current findings, for it may add to the evidence that prediabetes is not a benign state.^40^ Additionally, this would indicate that the window of opportunity for the prevention of brain disease in diabetes could be provided by prediabetes.

In conclusion, the available evidence indicates direct association of prediabetes or continuous HbA_1c_ and structural brain abnormalities risk. Thus, prevention and screening of structural brain abnormalities should begin in prediabetes stages. High‐quality, longitudinal and age‐related studies are needed to improve our understanding and cognition of this association.

## CONFLICT OF INTEREST

The authors declare no potential conflict of interest.

## AUTHOR CONTRIBUTION

The authors are solely responsible for the design and conduct of this study; all study analyses, the drafting and editing of the manuscript, and its final contents. X.Y.T., J.B.Z. and L.Q. have contributed to the design of the study. X.Y.T. and Y.P.H. analysis and interpretation of data, and prepared all figures and tables. X.Y.T., F.Q.L., Y.P.H. drafted a part of manuscript. F.Q.L., Y.P.H. took part in analysing data. All authors reviewed the manuscript. M.A.C. help review and correct English writing. All authors have read and approved the final manuscript.

## Supporting information


**Appendix** **S1.** Electronic supplementary material
