# Supplementary material for: Prediabetes and structural brain abnormalities: Evidence from observational studies
Source: Diabetes Metab Res Rev. 2019 Dec 19;36(4):e3261. doi: 10.1002/dmrr.3261 (PMC7685098; doi:10.1002/dmrr.3261)
Supplement: Supplementary file 1 — Appendix S1. Electronic supplementary material [file DMRR-36-e3261-s001.docx]

**Electronic supplementary material**

**Appendix.** Search Strategies

**Table 1.** Structural brain abnormalities and outcome definitions.

**Table 2.** Data of meta-analysis.

**Table 3.** Data of continuous structural brain abnormalities synthesized for meta-analysis.

**Table 4.** Risk of bias assessment (Newcastle-Ottawa Quality Assessment Scale criteria).

**Figure 1.** Cerebral microbleeds and prediabetes.

**Figure 2.** Grey matter volume and prediabetes.

**Figure 3.** Hippocampal volume and prediabetes.

**Figure 4.** Continuous total brain volume and prediabetes.

**Figure 5.** Funnel plot for prediabetes and infarct.

**Appendix.** Search Strategies

The search was undertaken on April, 1 2019.

The search was conducted through in the PubMed, Embase, Medline and Web of Science.

Search terms were as follows:

1. Embase

('glucose blood level' OR 'hemoglobin a1c' OR 'blood fluctuation' OR 'impaired glucose tolerance') AND 'white matter hyperintensities' AND 'nuclear magnetic resonance imaging'

('glucose blood level' OR 'hemoglobin a1c' OR 'blood fluctuation' OR 'impaired glucose tolerance') AND 'brain atrophy' AND 'nuclear magnetic resonance imaging'

('glucose blood level' OR 'hemoglobin a1c' OR 'blood fluctuation' OR 'impaired glucose tolerance') AND 'brain hemorrhage' AND 'nuclear magnetic resonance imaging'

('glucose blood level' OR 'hemoglobin a1c' OR 'blood fluctuation' OR 'impaired glucose tolerance') AND 'lacunar stroke' AND 'nuclear magnetic resonance imaging'

('glucose blood level' OR 'hemoglobin a1c' OR 'blood fluctuation' OR 'impaired glucose tolerance') AND 'lacunar infarct' AND 'nuclear magnetic resonance imaging'

('glucose blood level' OR 'hemoglobin a1c' OR 'blood fluctuation' OR 'impaired glucose tolerance') AND 'total cerebral brain volume' AND 'nuclear magnetic resonance imaging'

('glucose blood level' OR 'hemoglobin a1c' OR 'blood fluctuation' OR 'impaired glucose tolerance') AND 'white matter hyperintensity volume' AND 'nuclear magnetic resonance imaging'

('glucose blood level' OR 'hemoglobin a1c' OR 'blood fluctuation' OR 'impaired glucose tolerance') AND 'brain infarction' AND 'nuclear magnetic resonance imaging'

2. Pubmed

((blood glucose[MeSH Terms]) OR blood glucose) OR ((hemoglobin a1c[MeSH Terms]) OR hemoglobin a1c) OR ((blood fluctuation[MeSH Terms]) OR blood fluctuation) OR ((prediabetes[MeSH Terms]) OR prediabetes) OR ((impaired glucose tolerance[MeSH Terms]) OR impaired glucose tolerance) AND ((white matter hyperintensities[MeSH Terms]) OR white matter hyperintensities) AND ((Magnetic Resonance Imaging[MeSH Terms]) OR Magnetic Resonance Imaging)

((blood glucose[MeSH Terms]) OR blood glucose) OR ((hemoglobin a1c[MeSH Terms]) OR hemoglobin a1c) OR ((blood fluctuation[MeSH Terms]) OR blood fluctuation) OR ((prediabetes[MeSH Terms]) OR prediabetes) OR ((impaired glucose tolerance[MeSH Terms]) OR impaired glucose tolerance) AND ((brain atrophy [MeSH Terms]) OR brain atrophy) AND ((Magnetic Resonance Imaging[MeSH Terms]) OR Magnetic Resonance Imaging)

((blood glucose[MeSH Terms]) OR blood glucose) OR ((hemoglobin a1c[MeSH Terms]) OR hemoglobin a1c) OR ((blood fluctuation[MeSH Terms]) OR blood fluctuation) OR ((prediabetes[MeSH Terms]) OR prediabetes) OR ((impaired glucose tolerance[MeSH Terms]) OR impaired glucose tolerance) AND ((brain hemorrhage [MeSH Terms]) OR brain hemorrhage) AND ((Magnetic Resonance Imaging[MeSH Terms]) OR Magnetic Resonance Imaging)

((blood glucose[MeSH Terms]) OR blood glucose) OR ((hemoglobin a1c[MeSH Terms]) OR hemoglobin a1c) OR ((blood fluctuation[MeSH Terms]) OR blood fluctuation) OR ((prediabetes[MeSH Terms]) OR prediabetes) OR ((impaired glucose tolerance[MeSH Terms]) OR impaired glucose tolerance) AND ((cerebral microbleed[MeSH Terms]) OR cerebral microbleed) AND ((Magnetic Resonance Imaging[MeSH Terms]) OR Magnetic Resonance Imaging)

((blood glucose[MeSH Terms]) OR blood glucose) OR ((hemoglobin a1c[MeSH Terms]) OR hemoglobin a1c) OR ((blood fluctuation[MeSH Terms]) OR blood fluctuation) OR ((prediabetes[MeSH Terms]) OR prediabetes) OR ((impaired glucose tolerance[MeSH Terms]) OR impaired glucose tolerance) AND ((lacunar stroke[MeSH Terms]) OR lacunar stroke) AND ((Magnetic Resonance Imaging[MeSH Terms]) OR Magnetic Resonance Imaging)

((blood glucose[MeSH Terms]) OR blood glucose) OR ((hemoglobin a1c[MeSH Terms]) OR hemoglobin a1c) OR ((blood fluctuation[MeSH Terms]) OR blood fluctuation) OR ((prediabetes[MeSH Terms]) OR prediabetes) OR ((impaired glucose tolerance[MeSH Terms]) OR impaired glucose tolerance) AND ((lacunar infarct[MeSH Terms]) OR lacunar infarct) AND ((Magnetic Resonance Imaging[MeSH Terms]) OR Magnetic Resonance Imaging)

((blood glucose[MeSH Terms]) OR blood glucose) OR ((hemoglobin a1c[MeSH Terms]) OR hemoglobin a1c) OR ((blood fluctuation[MeSH Terms]) OR blood fluctuation) OR ((prediabetes[MeSH Terms]) OR prediabetes) OR ((impaired glucose tolerance[MeSH Terms]) OR impaired glucose tolerance) AND ((total cerebral brain volume[MeSH Terms]) OR total cerebral brain volume) AND ((Magnetic Resonance Imaging[MeSH Terms]) OR Magnetic Resonance Imaging)

((blood glucose[MeSH Terms]) OR blood glucose) OR ((hemoglobin a1c[MeSH Terms]) OR hemoglobin a1c) OR ((blood fluctuation[MeSH Terms]) OR blood fluctuation) OR ((prediabetes[MeSH Terms]) OR prediabetes) OR ((impaired glucose tolerance[MeSH Terms]) OR impaired glucose tolerance) AND ((hippocampal hyperintensity volume[MeSH Terms]) OR hippocampal hyperintensity volume) AND ((Magnetic Resonance Imaging[MeSH Terms]) OR Magnetic Resonance Imaging)

((blood glucose[MeSH Terms]) OR blood glucose) OR ((hemoglobin a1c[MeSH Terms]) OR hemoglobin a1c) OR ((blood fluctuation[MeSH Terms]) OR blood fluctuation) OR ((prediabetes[MeSH Terms]) OR prediabetes) OR ((impaired glucose tolerance[MeSH Terms]) OR impaired glucose tolerance) AND ((white matter hyperintensity volume[MeSH Terms]) OR white matter hyperintensity volume) AND ((Magnetic Resonance Imaging[MeSH Terms]) OR Magnetic Resonance Imaging)

((blood glucose[MeSH Terms]) OR blood glucose) OR ((hemoglobin a1c[MeSH Terms]) OR hemoglobin a1c) OR ((blood fluctuation[MeSH Terms]) OR blood fluctuation) OR ((prediabetes[MeSH Terms]) OR prediabetes) OR ((impaired glucose tolerance[MeSH Terms]) OR impaired glucose tolerance) AND ((brain infarct[MeSH Terms]) OR brain infarct) AND ((Magnetic Resonance Imaging[MeSH Terms]) OR Magnetic Resonance Imaging)

((blood glucose[MeSH Terms]) OR blood glucose) OR ((hemoglobin a1c[MeSH Terms]) OR hemoglobin a1c) OR ((blood fluctuation[MeSH Terms]) OR blood fluctuation) OR ((prediabetes[MeSH Terms]) OR prediabetes) OR ((impaired glucose tolerance[MeSH Terms]) OR impaired glucose tolerance) AND ((brain infarction[MeSH Terms]) OR brain infarction) AND ((Magnetic Resonance Imaging[MeSH Terms]) OR Magnetic Resonance Imaging)

3. Medline：

((((((blood glucose) OR hemoglobin a1c) OR blood fluctuation) OR prediabetes) OR impaired glucose tolerance) AND white matter hyperintensities) AND Magnetic Resonance Imaging

((((((blood glucose) OR hemoglobin a1c) OR blood fluctuation) OR prediabetes) OR impaired glucose tolerance) AND brain atrophy) AND Magnetic Resonance Imaging

((((((blood glucose) OR hemoglobin a1c) OR blood fluctuation) OR prediabetes) OR impaired glucose tolerance) AND brain hemorrhage) AND Magnetic Resonance Imaging

((((((blood glucose) OR hemoglobin a1c) OR blood fluctuation) OR prediabetes) OR impaired glucose tolerance) AND cerebral microbleed) AND Magnetic Resonance Imaging

((((((blood glucose) OR hemoglobin a1c) OR blood fluctuation) OR prediabetes) OR impaired glucose tolerance) AND lacunar stroke) AND Magnetic Resonance Imaging

((((((blood glucose) OR hemoglobin a1c) OR blood fluctuation) OR prediabetes) OR impaired glucose tolerance) AND lacunar infarct) AND Magnetic Resonance Imaging

((((((blood glucose) OR hemoglobin a1c) OR blood fluctuation) OR prediabetes) OR impaired glucose tolerance) AND total cerebral brain volume) AND Magnetic Resonance Imaging

((((((blood glucose) OR hemoglobin a1c) OR blood fluctuation) OR prediabetes) OR impaired glucose tolerance) AND hippocampal hyperintensity volume) AND Magnetic Resonance Imaging

((((((blood glucose) OR hemoglobin a1c) OR blood fluctuation) OR prediabetes) OR impaired glucose tolerance) AND white matter hyperintensity volume) AND Magnetic Resonance Imaging

((((((blood glucose) OR hemoglobin a1c) OR blood fluctuation) OR prediabetes) OR impaired glucose tolerance) AND brain infarct) AND Magnetic Resonance Imaging

((((((blood glucose) OR hemoglobin a1c) OR blood fluctuation) OR prediabetes) OR impaired glucose tolerance) AND brain infarction) AND Magnetic Resonance Imaging

4. Web of Science

((((((blood glucose) OR hemoglobin a1c) OR blood fluctuation) OR prediabetes) OR impaired glucose tolerance) AND white matter hyperintensities) AND Magnetic Resonance Imaging

((((((blood glucose) OR hemoglobin a1c) OR blood fluctuation) OR prediabetes) OR impaired glucose tolerance) AND brain atrophy) AND Magnetic Resonance Imaging

((((((blood glucose) OR hemoglobin a1c) OR blood fluctuation) OR prediabetes) OR impaired glucose tolerance) AND brain hemorrhage) AND Magnetic Resonance Imaging

((((((blood glucose) OR hemoglobin a1c) OR blood fluctuation) OR prediabetes) OR impaired glucose tolerance) AND cerebral microbleed) AND Magnetic Resonance Imaging

((((((blood glucose) OR hemoglobin a1c) OR blood fluctuation) OR prediabetes) OR impaired glucose tolerance) AND lacunar stroke) AND Magnetic Resonance Imaging

((((((blood glucose) OR hemoglobin a1c) OR blood fluctuation) OR prediabetes) OR impaired glucose tolerance) AND lacunar infarct) AND Magnetic Resonance Imaging

((((((blood glucose) OR hemoglobin a1c) OR blood fluctuation) OR prediabetes) OR impaired glucose tolerance) AND total cerebral brain volume) AND Magnetic Resonance Imaging

((((((blood glucose) OR hemoglobin a1c) OR blood fluctuation) OR prediabetes) OR impaired glucose tolerance) AND hippocampal hyperintensity volume) AND Magnetic Resonance Imaging

((((((blood glucose) OR hemoglobin a1c) OR blood fluctuation) OR prediabetes) OR impaired glucose tolerance) AND white matter hyperintensity volume) AND Magnetic Resonance Imaging

((((((blood glucose) OR hemoglobin a1c) OR blood fluctuation) OR prediabetes) OR impaired glucose tolerance) AND brain infarct) AND Magnetic Resonance Imaging

((((((blood glucose) OR hemoglobin a1c) OR blood fluctuation) OR prediabetes) OR impaired glucose tolerance) AND brain infarction) AND Magnetic Resonance Imaging

**Table 1.** Structural brain abnormalities and outcome definitions.

| study | year | structural brain disease | data |
| --- | --- | --- | --- |
| Hirabayashi et al | 2016 | total brain volume (TBV) intracranial volume (ICV) hippocampal volume (HV) | 1. IFG: No. of subjects: 53 TBV-to-ICV ratio (%): indicator of global brain atrophy: 78.7 (78.2-79.1) HV-to-ICV ratio (%): indicator of hippocampal atrophy: 0.533 (0.517-0.549) HV-to-TBV ratio (%): indicator of hippocampal atrophy beyond global brain atrophy: 0.677 (0.658-0.697) 2. IGT: No. of subjects: 280 TBV-to-ICV ratio (%): indicator of global brain atrophy: 78.5 (78.3-78.7) HV-to-ICV ratio (%): indicator of hippocampal atrophy: 0.537 (0.530-0.544) HV-to-TBV ratio (%): indicator of hippocampal atrophy beyond global brain atrophy: 0.684 (0.675-0.692) 3. FPG levels (mmol/L) 6.1-6.9: No. of subjects: 186 TBV-to-ICV ratio (%): indicator of global brain atrophy: 78.4 (78.1-78.6) HV-to-ICV ratio (%): indicator of hippocampal atrophy: 0.528 (0.519-0.536) HV-to-TBV ratio (%): indicator of hippocampal atrophy beyond global brain atrophy: 0.673 (0.662-0.683) 4. 2-h PG levels (mmol/L) 7.8-11.0: No. of subjects: 319 TBV-to-ICV ratio (%): indicator of global brain atrophy: 78.5 (78.3-78.6) HV-to-ICV ratio (%): indicator of hippocampal atrophy: 0.537 (0.530-0.543) HV-to-TBV ratio (%): indicator of hippocampal atrophy beyond global brain atrophy: 0.684 (0.676-0.692) |
| Imano et al | 2018 | total stroke hemorrhagic stroke ischemic stroke lacunar infarction | 1. total stroke (1) prediabetic type: No. of events: 49 Age-adjusted HR (95% CI): 1.94 (1.42-2.65) Multivariable HR (95% CI): 1.84 (1.33-2.55) (2) HR per 1 SD increment of glucose: No. of events: 291 Age-adjusted HR (95% CI): 1.19 (1.10-1.30) Multivariable HR (95% CI): 1.13 (1.03-1.24) 2. hemorrhagic stroke (1) prediabetic type: No. of events: 15 Age-adjusted HR (95% CI): 1.90 (1.08-3.33) Multivariable HR (95% CI): 1.64 (0.91-2.93) (2) HR per 1 SD increment of glucose: No. of events: 95 Age-adjusted HR (95% CI): 1.04 (0.86-1.27) Multivariable HR (95% CI): 0.93 (0.75-1.16) 3. ischemic stroke (1) prediabetic type: No. of events: 33 Age-adjusted HR (95% CI): 1.98 (1.35-2.91) Multivariable HR (95% CI): 1.96 (1.31-2.94) (2) HR per 1 SD increment of glucose: No. of events: 190 Age-adjusted HR (95% CI): 1.25 (1.14-1.38) Multivariable HR (95% CI): 1.22 (1.10-1.35) 4. lacunar infarction (1) prediabetic type: No. of events: 19 Age-adjusted HR (95% CI): 2.08 (1.25-3.47) Multivariable HR (95% CI): 2.02 (1.19-3.43) (2) HR per 1 SD increment of glucose: No. of events: 109 Age-adjusted HR (95% CI): 1.34 (1.20-1.49) Multivariable HR (95% CI): 1.29 (1.15-1.45) |

**Table 1.** Continued.

| study | year | structural brain disease | Data |
| --- | --- | --- | --- |
| Jin et al | 2019 | intracerebral hemorrhage | FBG 6.10-6.99 mmol/L Age- and sex- adjusted hazard ratio: 1.55 (1.20-1.99) Multivariate-adjusted hazard ratio 1: 1.56 (1.21-2.00) Multivariate-adjusted hazard ratio 2: 1.31 (1.02-1.69) |
| Walsh et al | 2018 | total brain volume grey matter volume thalamus volume white matter volume corpus callosum volume | 1. NFG (mean SD) total brain volume (mm^3^): 1124.94 (112.72) grey matter volume (mm^3^): 590.22 (55.2) thalamus volume (mm^3^): 15.62 (1.62) white matter volume (mm^3^): 505.14 (61.37) corpus callosum volume (mm^3^): 2.91 (0.55) 2. IFG (mean SD) total brain volume (mm^3^): 1123.92 (105.95) grey matter volume (mm^3^): 589.96 (52.06) thalamus volume (mm^3^): 15.61 (1.58) white matter volume (mm^3^): 504.35 (57.26) corpus callosum volume (mm^3^): 2.81 (0.44) |
| Marseglia et al | 2019 | TBTV: total brain tissue volume GMV: gray matter volume HV: hippocampal volume WMV: white matter volume WMHV: white matter hyperintensities volume | prediabetes β (95% CI) TBTV: -19.1 (-34.0 to -4.19) GMV: -9.07 (-19.5 to 1.33) HV: -0.06 (-0.22 to 0.11) WMV: -9.92 (-19.4 to -0.46) WMHV: 1.74 (-0.15 to 3.64) |
| Enzinger et al | 2005 | BPF: brain parenchymal fraction aBVC: annual brain volume change | 1. BPF (mean SD) HbA_1c_, % 4.4-5.2: 0.813 (0.141) HbA_1c_, % 5.3-5.5: 0.803 (0.019) HbA_1c_, % 5.6-5.8: 0.801 (0.023) HbA_1c_, % 5.9-9.0: 0.803 (0.016) 2.% aBVC (mean SD) HbA_1c_, % 4.4-5.2: -0.241 (0.169) HbA_1c_, % 5.3-5.5: -0.374 (0.331) HbA_1c_, % 5.6-5.8: -0.467 (0.288) HbA_1c_, % 5.9-9.0: -0.474 (0.271) 3. HbA_1c_ predicting subsequent aBVC, β (95% CI) Model 1: -0.093 (-0.161 to -0.024) Model 2: -0.093 (-0.160 to -0.025) Model 3: -0.087 (-0.150 to -0.025) |

**Table 1.** Continued.

| study | year | structural brain disease | data |
| --- | --- | --- | --- |
| Agtmaal et al | 2018 | 1. Markers of cerebral small-vessel disease lacunar infarcts (LIs) present (%) total white matter hyperintensities (WMH) (ml) deep cortical WMH (dWMH) (ml) periventricular WHMs (pWMH) (ml) cerebral microbleeds (CMBs) present (%) 2. Brain volumes (ml) White matter Grey matter cerebralspinal fluid (CSF) Intracranial | 1. NGM (1) Markers of cerebral small-vessel disease (interquartile range) LIs present (%): 4.1 total WMH (ml): 0.17 (0.05-0.52) dWMH (ml): 0.04 (0.01-0.15) pWMH (ml): 0.20 (0.05-0.73) CMBs present (%): 10.9 (2) Brain volumes (ml) (mean SD) White matter: 479.6 (59.5) Grey matter: 666.6 (58.8) CSF: 247.2 (45.8) Intracranial: 1394.1 (133.9) 2. prediabetes (1) Markers of cerebral small-vessel disease (interquartile range) LIs present (%): 7.9 total WMH (ml): 0.27 (0.07-1.12) dWMH (ml): 0.08 (0.01-0.30) pWMH (ml): 0.30 (0.09-0.90) CMBs present (%): 12.3 (2) Brain volumes (ml) (mean SD) White matter: 468.3 (62.6) Grey matter: 654.3 (62.9) CSF: 257.2 (50.0) Intracranial: 1381.1 (146.2) |
|  |  |  | 3. prediabetes (1) Markers of cerebral small-vessel disease LIs (yes/no), OR (95%CI): Model 1: 1.62 (1.00-2.64); Model 2: 1.61 (0.98-2.63) total WMH volume (log-ml), β (95% CI): Model 1: 0.08 (0.00-0.15); Model 2: 0.07 (0.00-0.15) dWMH volume (log-ml), β (95% CI): Model 1: 0.08 (0.00-0.16); Model 2: 0.07 (-0.01-0.15) pWMH volume (log-ml), β (95% CI): Model 1: 0.07 (0.00-0.14); Model 2: 0.06 (-0.01-0.13) CMBs (yes/no), OR (95%CI): Model 1: 0.85 (0.57-1.26); Model 2: 0.85 (0.57-1.27) (2) Brain volumes White matter volume (ml), β (95% CI): Model 1: -3.2 (-6.5 to 0.1); Model 2: -4.0 (-7.3 to -0.6) Grey matter volume (ml), β (95% CI): Model 1: -1.1 (-4.4 to 2.1); Model 2: -0.4 (-3.7 to 2.8) CSF (ml), β (95% CI): Model 1: 3.9 (0.3-7.6); Model 2: 3.9 (0.8-7.6) 4. HbA_1c_ (1) Markers of cerebral small-vessel disease LIs (yes/no), OR (95%CI): Model 1: 1.28 (1.11-1.49); Model 2: 1.28 (1.09-1.50) total WMH volume (log-ml), β (95% CI): Model 1: 0.10 (0.07-0.14); Model 2: 0.09 (0.05-0.13) dWMH volume (log-ml), β (95% CI): Model 1: 0.07 (0.03-0.11); Model 2: 0.06 (0.01-0.10) pWMH volume (log-ml), β (95% CI): Model 1: 0.11 (0.08-0.15); Model 2: 0.10 (0.06-0.14) CMBs (yes/no), OR (95%CI): Model 1: 1.05 (0.92-1.19); Model 2: 1.04 (0.90-1.20) (2) Brain volumes White matter volume (ml), β (95% CI): Model 1: -0.02 (-0.04 to 0.00); Model 2: -0.02 (-0.04 to 0.00) Grey matter volume (ml), β (95% CI): Model 1: -0.05 (-0.07 to -0.04); Model 2: -0.04 (-0.06 to -0.02) CSF (ml), β (95% CI): Model 1: 0.09 (0.06-0.11); Model 2: 0.07 (0.05-0.10) |

**Table 1.** Continued.

| study | year | structural brain disease | data |
| --- | --- | --- | --- |
| Schneider et al | 2017 | 1. Mean volume total brain frontal lobe temporal lobe occipital lobe parietal lobe deep gray matter: thalamus + putamen + caudate + globus pallidus Alzheimer disease signature region: hippocampus + parahippocampal + entorhinal + inferioe parietal lobule + precuneus + cuneus hippocampus 2. Markers of subclinecal cerebrovascular disease lobar microhemorrhages subcortical microhemorrhages cortical infarcts lacunar infarcts 3. Median WMH volume | 1. no diabetes (HbA1c<5.7%) (1) Mean volume (cm^3^) total brain: 1030.1 frontal lobe: 152.3 temporal lobe: 103.8 occipital lobe: 41.7 parietal lobe: 108.4 deep gray matter: 30.1 Alzheimer disease signature region: 60.5 hippocampus: 7.0 (2) Markers of subclinecal cerebrovascular disease (%) lobar microhemorrhages: 6.2 subcortical microhemorrhages: 17.8 cortical infarcts: 7.2 lacunar infarcts: 14.4 (3) Median WMH volume (cm^3^): 9.7 2. prediabetes (HbA1c 5.7 to 6.5%) (1) Mean volume (cm^3^) (SD) total brain: 1027.8 (108.1) frontal lobe: 152.2 (16.0) temporal lobe: 103.6 (11.7) occipital lobe: 41.0 (5.5) parietal lobe: 107.7 (12.6) deep gray matter: 30.3 (4.3) Alzheimer disease signature region: 60.1 (7.0) hippocampus: 7.1 (1.0) (2) Markers of subclinecal cerebrovascular disease (%) lobar microhemorrhages: 9.9 subcortical microhemorrhages: 17.7 cortical infarcts: 10.8 lacunar infarcts: 16.8 (3) Median WMH volume (cm^3^): 10.2 |
|  |  |  | 3. prediabetes^l^ (HbA_1c_ 5.7 to 6.5%) (1) Volumes, β (95% CI) total brain: 0.01 (-0.05, 0.08) frontal lobe: 0.03 (-0.05, 0.11) temporal lobe: 0.02 (-0.06, 0.10) occipital lobe: -0.04 (-0.14, 0.06) parietal lobe: 0.02 (-0.06, 0.10) deep gray matter: 0.05 (-0.06, 0.16) Alzheimer disease signature region: 0.01 (-0.07, 0.09) hippocampus: 0.04 (-0.07, 0.15) (2) Markers of subclinecal cerebrovascular disease, OR (95% CI) lobar microhemorrhages: 1.62 (0.93, 2.80) subcortical microhemorrhages: 0.88 (0.60, 1.29) cortical infarcts: 1.44 (0.86, 2.41) lacunar infarcts: 1.10 (0.74, 1.62) (3) Log_2_ WMH volume, β (95% CI): 0.11 (-0.06, 0.29) |

**Table 1.** Continued.

| study | year | structural brain disease | data |
| --- | --- | --- | --- |
| Exalto et al | 2014 | NBV: normalized brain volume MTA: medial temporal lobe atrophy WMH: white matter hyperintensities lacunes | Relation between HbA_1c_, brain volumes and vascular lesions: NBV (ml), β (95%CI): unadjusted: -0.04 (-0.17-0.09); adjusted^k^: 0.09 (-0.03-0.20) MTA, OR (95% CI): unadjusted: 1.2 (0.97-1.5); adjusted^k^: 1.0 (0.8-1.4) WMH, OR (95% CI): unadjusted: 1.1 (0.8-1.5); adjusted^k^: 0.9 (0.7-1.3) Lacunes, OR (95% CI): unadjusted: 1.1 (0.8-1.4); adjusted^k^: 1.0 (0.7-1.3) |
| Reitz et al | 2016 | white matter hyperintensities total gray matter volume total white matter volume hippocampal volume intracranial volume infarcts | 1. NGT (SD) white matter hyperintensities: 7.6 (9.6) total gray matter volume: 553703.9 (61334.6) total white matter volume: 412060.9 (63956.0) hippocampal volume: 6784.6 (1043.0) intracranial volume: 1356590.6 (167875.7) 2. prediabetes (SD) white matter hyperintensities: 7.9 (9.7) total gray matter volume: 541412.2 (46802.3) total white matter volume: 400916.9 (53170.4) hippocampal volume: 6746.9 (854.4) intracranial volume: 1309562.3 (151701.5) 3. HbA_1c_ continuous presence of infarcts, OR (95% CI): Model 1: 1.03 (0.77-1.39); Model 2: 1.02 (0.75-1.37); Model 3: 1.01 (0.74-1.37); Model 4: 0.96 (0.68-1.34) number of infarcts, β: Model 1: 0.01; Model 2: 0.01; Model 3: 0.02; Model 4: -0.01 white matter hyperintensities, β: Model 1: 0.12; Model 2: 0.12; Model 3: 0.12; Model 4: 0.13 corical white matter volume, β: Model 1: -2272.14; Model 2: -2767.90; Model 3: -3015.86; Model 4: -3229.08 total gray volume, β: Model 1: -4076.14; Model 2: -4819.65; Model 3: -5016.86; Model 4: -6118.22 total hippocampus volume, β: Model 1: -18.96; Model 2: -32.87; Model 3: -38.64; Model 4: -61.28 4. prediabetes presence of infarcts, OR (95% CI): Model 1: 0.92 (0.56-1.50); Model 2: 0.90 (0.55-1.48); Model 3: 0.94 (0.56-1.56); Model 4: 0.79 (0.45-1.38) number of infarcts, β: Model 1: -0.06; Model 2: -0.07; Model 3: -0.03; Model 4: -0.01 white matter hyperintensities, β: Model 1: 0.08; Model 2: 0.07; Model 3: 0.08; Model 4: 0.09 corical white matter volume, β: Model 1: -1291.98; Model 2: -1106.48; Model 3: -255.21; Model 4: 137.05 total gray volume, β: Model 1: -1681.04; Model 2: -1411.10; Model 3: -1104.79; Model 4: -1846.03 total hippocampus volume, β: Model 1: 44.46; Model 2: 53.04; Model 3: 35.56; Model 4: -19.24 |

**Table 1.** Continued.

| study | year | structural brain disease | data |
| --- | --- | --- | --- |
| Saczynski et al | 2009 | total brain volume grey matter white matter WMLs: white matter lesion | 1. normoglycemic Brain volume (% of total intracranial volume) (SD) total brain volume: 72.25 (3.8) grey matter: 45.18 (1.8) white matter: 25.74 (1.3) WMLs: 1.32 (3.3) 2. IFG Brain volume (% of total intracranial volume) (SD) total brain volume: 72.23 (3.9) grey matter: 45.23 (1.9) white matter: 25.71 (1.3) WMLs: 1.29 (3.2) 3. IFG, OR (95% CI) Model 1: single infarct, 1.00 (0.85, 1.17) Model 2: single infarct, 0.97 (0.82, 1.14) |
| Eastwood et al | 2015 | cerebrovascular disease (stroke or brain infarct≥3mm) | prediabetes, OR (95% CI) 1. European group OGTT: Model 1: 1.42 (0.90-2.25); Model 2: 1.25 (0.63-1.88) HbA_1c_: Model 1: 1.73 (1.10-2.72); Model 2: 1.57 (1.00-2.51) 2. South Asian group OGTT: Model 1: 0.91 (0.45-1.86); Model 2: 1.02 (0.49-4.44) HbA_1c_: Model 1: 1.41 (0.72-2.76); Model 2: 1.39 (0.69-2.78) |

**Abbreviations:** HR, hazard ratio; OR, odds ratio; SD, standard deviation; IGT, impaired glucose tolerance; IFG, impaired fasting glucose; NFG, normal fasting glucose; HbA_1c_, glycosylate hemoglobin.

**Table 2.** Data of meta-analysis.

| study | year | type | structural brain abnormalities |  | OR | ll | ul | β | ul | ll |
| --- | --- | --- | --- | --- | --- | --- | --- | --- | --- | --- |
| Reitz et al | 2016 | prediabetes | infarct | presence of infarcts | 0.92 | 0.56 | 1.5 |  |  |  |
| Eastewood et al | 2015 | prediabetes | infarct | cerebrovascular disease (stroke or brain infarct ≥ 3mm) European group OGTT | 1.42 | 0.9 | 2.25 |  |  |  |
| Eastewood et al | 2015 | prediabetes | infarct | cerebrovascular disease (stroke or brain infarct ≥ 3mm) European group HbA_1c_ | 1.73 | 1.1 | 2.72 |  |  |  |
| Eastewood et al | 2015 | prediabetes | infarct | cerebrovascular disease (stroke or brain infarct ≥ 3mm) South Asian group OGTT | 0.91 | 0.45 | 1.86 |  |  |  |
| Eastewood et al | 2015 | prediabetes | infarct | cerebrovascular disease (stroke or brain infarct ≥ 3mm) South Asian group HbA_1c_ | 1.41 | 0.72 | 2.76 |  |  |  |
| Schneider et al | 2017 | prediabetes | infarct | cortical infarcts | 1.44 | 0.86 | 2.41 |  |  |  |
| Schneider et al | 2017 | prediabetes | infarct | lacunar infarcts | 1.1 | 0.74 | 1.62 |  |  |  |
| Agtmaal et al | 2018 | prediabetes | infarct | lacunar infarcts present | 1.62 | 1 | 2.64 |  |  |  |
| Saczynski et al | 2009 | prediabetes | infarct | single infarct | 1 | 0.85 | 1.17 |  |  |  |
| Marseglia et al | 2019 | prediabetes | white matter hyperintensities | white matter hyperintensities volume |  |  |  | 1.74 | -0.15 | 3.64 |
| Schneider et al | 2017 | prediabetes | white matter hyperintensities | Log_2_ WMH volume |  |  |  | 0.11 | -0.06 | 0.29 |
| Agtmaal et al | 2018 | prediabetes | white matter hyperintensities | total (log-ml) |  |  |  | 0.08 | 0 | 0.15 |
| Agtmaal et al | 2018 | prediabetes | white matter hyperintensities | dWMH (log-ml) |  |  |  | 0.08 | 0 | 0.16 |
| Agtmaal et al | 2018 | prediabetes | white matter hyperintensities | pWMH (log-ml) |  |  |  | 0.07 | 0 | 0.14 |
| Marseglia et al | 2019 | prediabetes | hippocampal volume | hippocampal volume |  |  |  | -0.06 | -0.22 | 0.11 |
| Schneider et al | 2017 | prediabetes | hippocampal volume | hippocampal volume |  |  |  | 0.04 | -0.07 | 0.15 |
| Marseglia et al | 2019 | prediabetes | white matter volume | white matter volume |  |  |  | -9.92 | -19.4 | -0.46 |

**Table 2.** Continued.

| study | year | type | structural brain abnormalities |  | OR | ll | ul | β | ul | ll |
| --- | --- | --- | --- | --- | --- | --- | --- | --- | --- | --- |
| Agtmaal et al | 2018 | prediabetes | white matter volume | white matter volume |  |  |  | -3.2 | -6.5 | 0.1 |
| Marseglia et al | 2019 | prediabetes | grey matter volume | grey matter volume |  |  |  | -9.07 | -19.5 | 1.33 |
| Schneider et al | 2017 | prediabetes | grey matter volume | deep gray matter volume |  |  |  | 0.05 | -0.06 | 0.16 |
| Agtmaal et al | 2018 | prediabetes | grey matter volume | grey matter volume |  |  |  | -1.1 | -4.4 | 2.1 |
| Marseglia et al | 2019 | prediabetes | total brain volume | total brain tissue volume |  |  |  | -19.1 | -34 | -4.19 |
| Schneider et al | 2017 | prediabetes | total brain volume | total brain volume |  |  |  | 0.01 | -0.05 | 0.08 |
| Agtmaal et al | 2018 | prediabetes | cerebral microbleeds | cerebral microbleeds | 0.85 | 0.57 | 1.26 |  |  |  |
| Schneider et al | 2017 | prediabetes | cerebral microbleeds | lobar microhemorrhages | 1.62 | 0.93 | 2.8 |  |  |  |
| Schneider et al | 2017 | prediabetes | cerebral microbleeds | subcortical microhemorrhages | 0.88 | 0.6 | 1.29 |  |  |  |
| Exalto et al | 2014 | HbA_1c_ | white matter hyperintensities | adjusted | 0.9 | 0.7 | 1.3 |  |  |  |
| Agtmaal et al | 2018 | HbA_1c_ | white matter hyperintensities | total (log-ml) Model 1 |  |  |  | 0.1 | 0.07 | 0.14 |
| Agtmaal et al | 2018 | HbA_1c_ | white matter hyperintensities | dWMH (log-ml) Model 1 |  |  |  | 0.07 | 0.03 | 0.11 |
| Agtmaal et al | 2018 | HbA_1c_ | white matter hyperintensities | pWMH (log-ml) Model 1 |  |  |  | 0.11 | 0.08 | 0.15 |
| Exalto et al | 2014 | HbA_1c_ | infarct | lacunes adjusted | 1 | 0.7 | 1.3 |  |  |  |
| Reitz et al | 2016 | HbA_1c_ | infarct | presence of infarcts Model 1 | 1.03 | 0.77 | 1.39 |  |  |  |
| Agtmaal et al | 2018 | HbA_1c_ | infarct | lacunar infarcts present Model 1 | 1.28 | 1.11 | 1.49 |  |  |  |
| Enzinger et al | 2005 | HbA_1c_ | brain volume | annual brain volume Model 1 |  |  |  | -0.093 | -0.161 | -0.024 |
| Exalto et al | 2014 | HbA_1c_ | brain volume | normalized brain volume adjusted |  |  |  | 0.09 | -0.03 | 0.2 |

**Abbreviations:** OR, odd ratios; ll, lower limit of 95% confidence interval; ul, upper limit of 95% confidence interval; OGTT, oral glucose tolerance test; HbA1c, glycosylated hemoglobin; WMH, white matter hyperintensities.

**Table 3.** Data of continuous structural brain abnormalities synthesized for meta-analysis.

| study | year | structural brain abnormalities | | ne | meane | sde | nc | meanc | sdc |
| --- | --- | --- | --- | --- | --- | --- | --- | --- | --- |
| Reitz et al | 2016 | white matter volume | total white matter volume | 224 | 400.9169 | 53.1704 | 115 | 412.0609 | 63.956 |
| Agtmaal et al | 2018 | white matter volume | white matter volume | 347 | 468.3 | 62.6 | 1373 | 479.6 | 59.5 |
| Walsh et al | 2018 | white matter volume | white matter volume | 95 | 504.35 | 57.26 | 353 | 505.14 | 61.37 |
| Reitz et al | 2016 | grey matter volume | total gray matter volume | 224 | 541.4122 | 46.8023 | 115 | 553.7039 | 61.3346 |
| Agtmaal et al | 2018 | grey matter volume | gray matter volume | 347 | 654.3 | 62.9 | 1373 | 666.6 | 58.8 |
| Walsh et al | 2018 | grey matter volume | grey matter volume | 95 | 589.96 | 52.06 | 353 | 590.22 | 55.2 |
| Walsh et al | 2018 | total brain volume | total brain volume | 95 | 1123.92 | 105.95 | 353 | 1124.94 | 112.72 |
| Agtmaal et al | 2018 | total brain volume | intracranial volume | 347 | 1381.1 | 146.2 | 1373 | 1394.1 | 133.9 |

**Abbreviations:** ne, number of experimental group; meane, mean value of experimental group; sde, standard deviation of experimental group; nc, number of control group; meanc, mean value of control group; sdc, standard deviation of control group.

**Table 4.** Risk of bias assessment (Newcastle-Ottawa Quality Assessment Scale criteria).

| **Study** | **Study type** | **Selection** |  |  |  | **Comparability** | **Outcome** |  |  | **Quality  score** |
| --- | --- | --- | --- | --- | --- | --- | --- | --- | --- | --- |
|  |  | Is the case definition adequate? (cross-sectional) Representativeness of the exposed cohort (cohort) | Representativeness of the cases (cross-sectional) Selection of the non-exposed cohort (cohort) | Selection of controls from same sourse as the cases (cross-sectional) Ascertainment of exposure (cohort) | Definition of controls (cross-sectional) Demonstration that the outcome of interest was not present at start of the study (cohort) | Comparability of cases and controls on the basis of the design or the analysis | Ascertainment of exposure (cross-sectional) Ascertainment of outcome (cohort) | Same method of ascertainment for cases and controls? (cross-sectional) Was follow-up long enough for outcomes to occur? (cohort) | Non-response rate (cross-sectional) Adequacy of follow-up of cohorts (cohort) |  |
| Hirabayashi et al 2016 | cross-sectional study | Yes. Glucose tolerance status was defined according to the 1998 World Health Organization criteria.* | The Hisayama Study was established in 1961 in the town of Hisayama, a suburb of the Fukuoka metropolitan area of Kyushu Island in Japan.* | Yes.* | Normal glucose tolerance.* | Study controls for age.* | In the MRI examination, T1-weighted three-dimensional magnetizationprepared rapid gradient echo images, conventional T1- and T2-weighted images, fluid attenuated inversion recovery images, T2*-weighted images, and magnetic resonance angiography of the brain were examined using a 1.5-Tesla MRI scanner with amultichannel head coil. * | Yes.* | Same rate for both groups* | 8 |
| Walsh et al 2018 | cross-sectional study | Yes. Impaired fasting glucose was defined following American Diabetes Association guidelines.* | A large cohort of healthy community-living adults.* | Yes.* | Normal fasting glucose.* | Study controls for age.* | Using MRI and scans were three-dimensional structural fast-field echo sequence T1-weighted, analyzed using the longitudinal pipeline of Freesurfer v5.3 on a Linux workstation.* | Yes.* | Same rate for both groups* | 8 |
| Marseglia et al 2019 | cross-sectional study | Yes. HbA1c (%) was measured with the Swedish Mono-S filament High Performance Liquid Chromatography. Prediabetes was identified in nondiabetic participants with HbA1c levels of 5.7%–6.5%.* | The population-based study targeting people aged 60 years living at home or in institutions in central Stockholm. | Yes.* | Diabetes free.* | Study controls for age.* | Using MRI.* | Yes.* | Same rate for both groups* | 7 |
| Agtmaal et al 2018 | cross-sectional study | Yes. Venous fasting and postload plasma glucose levels were measured by the enzymatic hexokinase method on two automatic analyzers. HbA1c was determined by ion-exchange highperformance liquid chromatography.* | Used data from the Maastricht Study, an observational population-based cohort study. * | Yes.* | Normal glucose metabolism.* | Study controls for age.* | T2-weighted FLAIR and T1 images were used to identify WMHs. T1 images and T2-weighted FLAIR images were analyzed by use of an ISO-13485:2012–certified, automated method.* | Yes.* | Same rate for both groups* | 8 |
| Schneider et al 2017 | cross-sectional study | Yes. Whole-blood samples were assayed for HbA1c measurement by using high-performance liquid chromatography.* | The ARIC Study is an ongoing, communitybased prospective cohort study of 15,792 middle-aged adults recruited from four U.S. communities: Washington County, Maryland; Forsyth County, North Carolina; the suburbs of Minneapolis, Minnesota; and Jackson, Mississippi.* | Yes.* | No diabetes.* | Study controls for age.* | The ARIC visit 5 (2011–2013) brain MRI scans were performed by using four 3T scanners.* | Yes.* | Same rate for both groups* | 8 |

**Table 4.** Continued.

| **Study** | **Study type** | **Selection** |  |  |  | **Comparability** | **Outcome** |  |  | **Quality  score** |
| --- | --- | --- | --- | --- | --- | --- | --- | --- | --- | --- |
|  |  | Is the case definition adequate? (cross-sectional) Representativeness of the exposed cohort (cohort) | Representativeness of the cases (cross-sectional) Selection of the non-exposed cohort (cohort) | Selection of controls from same sourse as the cases (cross-sectional) Ascertainment of exposure (cohort) | Definition of controls (cross-sectional) Demonstration that the outcome of interest was not present at start of the study (cohort) | Comparability of cases and controls on the basis of the design or the analysis | Ascertainment of exposure (cross-sectional) Ascertainment of outcome (cohort) | Same method of ascertainment for cases and controls? (cross-sectional) Was follow-up long enough for outcomes to occur? (cohort) | Non-response rate (cross-sectional) Adequacy of follow-up of cohorts (cohort) |  |
| Reitz et al 2016 | cross-sectional study | Yes. HbA1C was measured by boronate affinity chromatography with the Primus CLC 385. “Dysglycemia” categories were defined based on HBA1C levels following American Diabetes Association guidelines.* | Participants were selected from a cohort participating in the prospective study of aging and dementia in Medicare recipients, 65 years and older and residing in northern Manhattan.* | Yes.* | Normal glucose tolerance.* | Study controls for age.* | Scan acquisition was performed on a 1.5T Philips Intera scanner at Columbia University Medical Center.* | Yes.* | Same rate for both groups* | 8 |
| Saczynski et al 2009 | cross-sectional study | Yes. Glycemic groups were defined using American Diabetes Association cut points.* | Participants are from the cohort of men and women born in 1907–1935 and living in Reykjavik.* | Yes.* | Normoglycemic.* | Study controls for age.* | High-resolution MRI scans were acquired on a 1.5-T Signa Twinspeed system. It includes the following pulse sequences: a proton density/T2-weighted fast spin echo sequence and a fluid-attenuated inversion recovery sequence.* | Yes.* | Same rate for both groups* | 8 |
| Eastwood et al 2015 | cross-sectional study | Yes. Glucose levels, lipid profile, HbA1c and C-reactive protein levels were measured on fasting blood samples, and anthropometry performed.* | Using data from the Southall and Brent Revisited (SABRE) study, a multi-ethnic population-based cohort of individuals living in north-west London.* | Yes.* | Normoglycemic.* | Study controls for age.* | Using an MRI scanning and scoring protocol based on that of the Cardiovascular Health Study. Whole-brain scans included sagittal T1-weighted images and axial T1-weighted, proton density and T2-weighted images of 5-mm thickness, with no gaps. Thin-section 3-mm axial fluid attenuated inversion recovery (FLAIR) and coronal 1.5-mm three-dimensional T1-weighted gradient echo images were also obtained. | Yes.* | Same rate for both groups* | 8 |
| Imano et al 2018 | cohort study | The participants were from 4 communities: Ikawa town, the Minami-Takayasu district in Yao City, Noichi town, and Kyowa town.* | Drawn from the same community as the exposed cohort* | Serum glucose was determined by the glucokinase method using an Autoanalyzer 7250 (Hitachi Medical Corp., Ibaraki, Japan).* | Yes.* | Study controls for age and history disease.* | The determination of stroke subtype was performed from the CT/MRI fndings according to our previous report.* | Yse.* | During the 14.1-yearfollow-up including 99,552 personyears, researchers documented 291 incidents of stroke (137 in men, 154 in women), 299 people who moved away from their baseline community (86 men, 213 women) and 1,085 deaths (614 men, 471 women).* | 8 |

**Table 4.** Continued.

| **Study** | **Study type** | **Selection** |  |  |  | **Comparability** | **Outcome** |  |  | **Quality  score** |
| --- | --- | --- | --- | --- | --- | --- | --- | --- | --- | --- |
|  |  | Is the case definition adequate? (cross-sectional) Representativeness of the exposed cohort (cohort) | Representativeness of the cases (cross-sectional) Selection of the non-exposed cohort (cohort) | Selection of controls from same sourse as the cases (cross-sectional) Ascertainment of exposure (cohort) | Definition of controls (cross-sectional) Demonstration that the outcome of interest was not present at start of the study (cohort) | Comparability of cases and controls on the basis of the design or the analysis | Ascertainment of exposure (cross-sectional) Ascertainment of outcome (cohort) | Same method of ascertainment for cases and controls? (cross-sectional) Was follow-up long enough for outcomes to occur? (cohort) | Non-response rate (cross-sectional) Adequacy of follow-up of cohorts (cohort) |  |
| Jin et al 2019 | cohort study | 96,110 participants of the Kailuan study, living in Kailuan community, Tangshan city, China.* | Drawn from the same community as the exposed cohort* | Fasting blood samples were collected in the morning after an 8- to 12-hour overnight fast and transfused into vacuum tubes containing EDTA (Ethylene Diamine Tetra Acetic acid).* | Yes.* | Study controls for age and history disease.* | With autopsy or CT/MRI imaging.* | Yse.* | Complete follow up all subject.* | 8 |
| Marseglia et al 2019 | cohort study | The population-based study targeting people aged 60 years living at home or in institutions in central Stockholm. | Drawn from the same community as the exposed cohort* | HbA1c (%) was measured with the Swedish Mono-S filament High Performance Liquid Chromatography. Prediabetes was identified in nondiabetic participants with HbA1c levels of 5.7%–6.5%.* | Yes.* | Study controls for age.* | Using MRI.* | Yes.* | Complete follow up all subject.* | 7 |
| Enzinger et al 2005 | cohort study | 201 participants in the Austrian Stroke Prevention Study.* | Drawn from the same community as the exposed cohort* | measured the plasma levels of fasting glucose and of the percentage of glycated hemoglobin A (HbA1c) of study participants. | Yes.* | Study controls for age and history disease.* | MRI was obtained on 1.5-T scanners from the same manufacturer.* | Yes.* | Complete follow up all subject.* | 7 |
| Exalto et al 2014 | cohort study | Atients were recruited from the outpatient memory clinic of the Alzheimer centre of the VU University Medical Centre (VUmc). | No stated. | Fasting glucose and HbA1c were determined in EDTA plasma at the Department of Clinical Chemistry of the VUmc.* | Yes.* | Study controls for age and history disease.* | Using MRI.* | Yes.* | Complete follow up all subject.* | 6 |
| Reitz et al 2016 | cohort study | Participants were selected from a cohort participating in the prospective study of aging and dementia in Medicare recipients, 65 years and older and residing in northern Manhattan.* | Drawn from the same community as the exposed cohort* | HbA1C was measured by boronate affinity chromatography with the Primus CLC 385. “Dysglycemia” categories were defined based on HBA1C levels following American Diabetes Association guidelines.* | Yes.* | Study controls for age.* | Scan acquisition was performed on a 1.5T Philips Intera scanner at Columbia University Medical Center.* | Yes.* | Complete follow up all subject.* | 8 |

**Figure 1.** The association between prediabetes with cerebral microbleeds (A), grey matter volume (B), hippocampal volume (C) and continuous total brain volume (D).


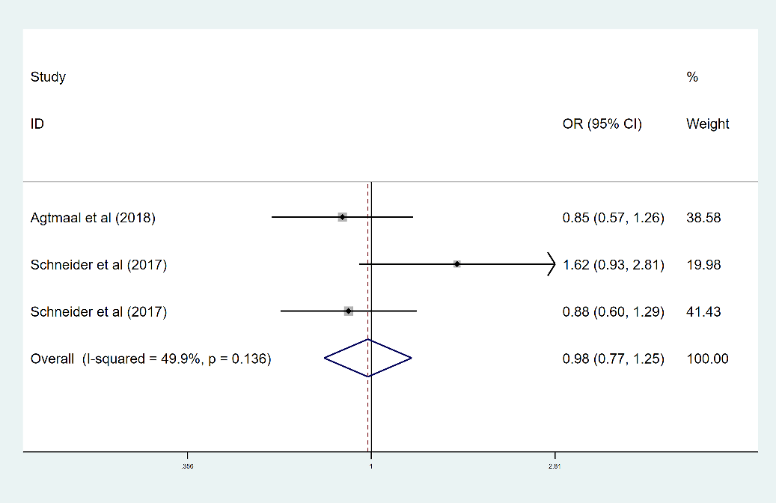

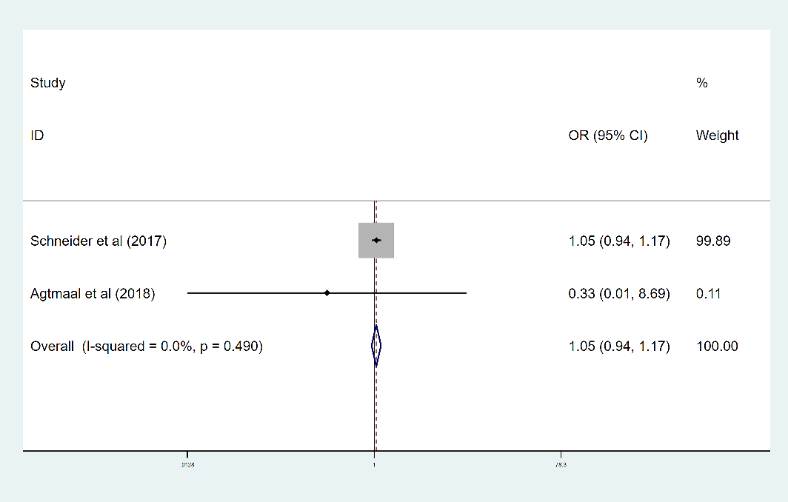


A. The association between prediabetes with cerebral microbleeds. B. The association between prediabetes with grey matter volume.


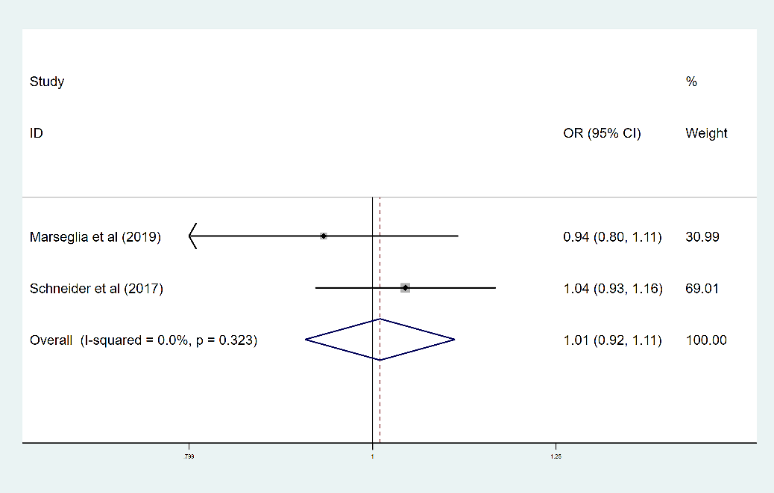

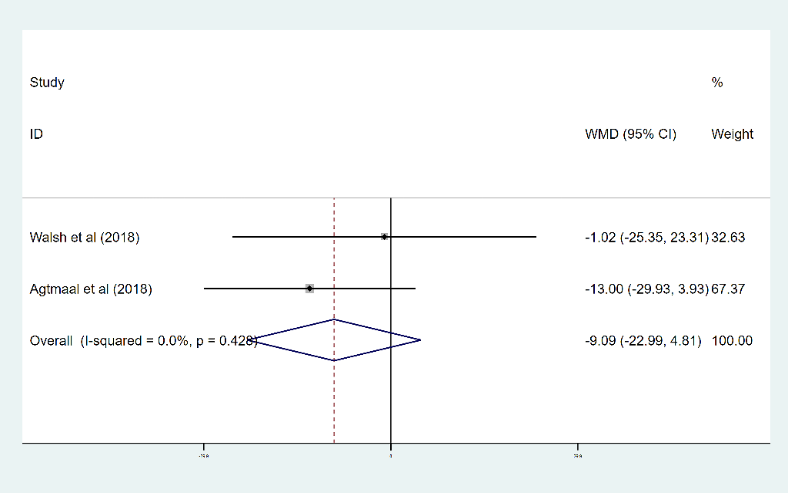


C. The association between prediabetes with hippocampal volume. D. The association between prediabetes with continuous total brain volume.

Where *I^2^* is the variation in effect estimates attributable to heterogeneity, overall is the pooled fixed effect estimate of all studies. subtotal is the pooled fixed effects estimate of sub-group analysis studies. Weights are from fixed-effects analysis. %Weight is the weight assigned to each study, based on the inverse of the within- and between-study variance. The size of the grey boxes around the point estimates reflects the weight assigned to each study. The summarized studies were adjusted for age, sex and BMI.

**Abbreviations:** OR, odd ratios.

**Figure 2.** Funnel plot for prediabetes and infarct.


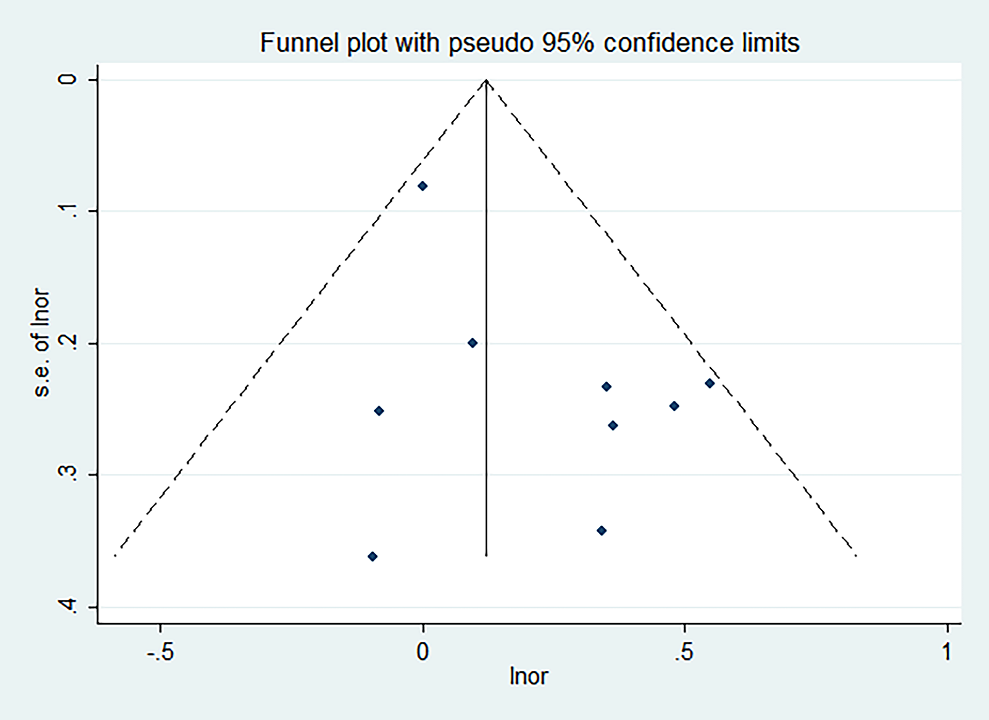


Each dot represents a different study. Asymmetry indicates smaller studies without statistically significant effects remain unpublished.
